# Comparative Modeling of Frequency Mixing Measurements of Magnetic Nanoparticles Using Micromagnetic Simulations and Langevin Theory

**DOI:** 10.3390/nano11051257

**Published:** 2021-05-11

**Authors:** Ulrich M. Engelmann, Ahmed Shalaby, Carolyn Shasha, Kannan M. Krishnan, Hans-Joachim Krause

**Affiliations:** 1 Department of Medical Engineering and Applied Mathematics, FH Aachen University of Applied Sciences, 52428 Jülich, Germany; ahmed.shalaby@alumni.fh-aachen.de; 2 Department of Physics, University of Washington, Seattle, WA 98195, USA; cshasha@uw.edu (C.S.); kannanmk@uw.edu (K.M.K.); 3 Department of Materials Science and Engineering, University of Washington, Seattle, WA 98195, USA; 4 Institute of Biological Information Processing—Bioelectronics (IBI-3), Forschungszentrum Jülich, 52425 Jülich, Germany

**Keywords:** magnetic nanoparticles, frequency mixing magnetic detection, Langevin theory, micromagnetic simulation, nonequilibrium dynamics, magnetic relaxation

## Abstract

Dual frequency magnetic excitation of magnetic nanoparticles (MNP) enables enhanced biosensing applications. This was studied from an experimental and theoretical perspective: nonlinear sum-frequency components of MNP exposed to dual-frequency magnetic excitation were measured as a function of static magnetic offset field. The Langevin model in thermodynamic equilibrium was fitted to the experimental data to derive parameters of the lognormal core size distribution. These parameters were subsequently used as inputs for micromagnetic Monte-Carlo (MC)-simulations. From the hysteresis loops obtained from MC-simulations, sum-frequency components were numerically demodulated and compared with both experiment and Langevin model predictions. From the latter, we derived that approximately 90% of the frequency mixing magnetic response signal is generated by the largest 10% of MNP. We therefore suggest that small particles do not contribute to the frequency mixing signal, which is supported by MC-simulation results. Both theoretical approaches describe the experimental signal shapes well, but with notable differences between experiment and micromagnetic simulations. These deviations could result from Brownian relaxations which are, albeit experimentally inhibited, included in MC-simulation, or (yet unconsidered) cluster-effects of MNP, or inaccurately derived input for MC-simulations, because the largest particles dominate the experimental signal but concurrently do not fulfill the precondition of thermodynamic equilibrium required by Langevin theory.

## 1. Introduction

Magnetic nanoparticles (MNPs) have a plethora of applications not only in biomedical diagnostics, mainly determined by sample preparation, but also in detection [1,2]. MNPs are used as markers for biomolecules in immunoassays; in addition to the well-established techniques of AC-Susceptometry [3] and Relaxometry [4], Frequency-Mixing Magnetic Detection (FMMD) [5] has been increasingly applied during the past decade because of its high selectivity. This technique relies on simultaneously applying a low-frequency magnetic driving field, which brings the particles close to saturation, and a high-frequency excitation field, which probes the particles’ susceptibility. Due to the nonlinear magnetization of the MNP, harmonics of both the individual incident frequencies and the intermodulation products of both frequencies are generated. Their signal can usually be picked up using a receive coil; however, other magnetic detectors can also be employed [6]. Due to its high sensitivity, FMMD has been successfully applied to realize magnetic immunoassays for the detection of a multitude of different analytes, for instance, viruses [7], antibiotics [8], or bacterial toxins [9]. Furthermore, FMMD enables the distinction of different types of MNP based on their different frequency mixing response spectrum [10,11,12], which opens the potential for multi-analyte detection.

In this work, we present offset-field-dependent FMMD measurements of MNPs and compare them quantitatively with two different modeling approaches: a simple Langevin-function-based thermal equilibrium model based on a lognormal size distribution, and a micromagnetic Monte Carlo (MC)-simulation method [13]. Although particles are immobilized in the experiment and can thus only relax according to Néel relaxation, the MC-simulation also includes Brownian relaxation. With this, micromagnetic MC-simulations provide new insight into the frequency mixing excitation of MNPs by modeling their non-equilibrium dynamics. For the Langevin model, the nature of relaxation is irrelevant. The core size distribution parameters derived from the Langevin model were used as starting values for the MC-simulation. As a consistency check, the MC results were again compared against the Langevin model. Prospects and limitations of both models are discussed, and suggestions for future research are developed.

## 2. Materials and Methods

### 2.1. Experimental Setup for Frequency Mixing Magnetic Detection

A custom-built measurement setup was employed for simultaneous sample excitation and demodulation signal detection. This comprises two excitation and two pick-up coils in a measurement head unit, allowing digital frequency demodulation directly from the detected signal. Details on the setup can be found in earlier works [5,14,15]. The applied alternating magnetic excitation field (AMF) was:

(1) $$H\left(t\right)=H_{0}+H_{1}\sin\left(2\pi f_{1}t\right)+H_{2}\sin\left(2\pi f_{2}t\right)$$

where $H_{0}$ denotes the static magnetic offset field, $H_{1}=1.29$ mT/*µ*_0_ is the magnetic field amplitude at high frequency $f_{1}=30,543\;\mathrm{Hz}$, and $H_{2}=16.4$ mT/*µ*_0_ is the amplitude at low frequency $f_{2}=62.95$ Hz. With this low-frequency amplitude, the particles are driven well into the nonlinear regime of the magnetization curve. The almost 400-fold higher *f*_1_ yields well-detectable voltages at the mixing frequencies.

ABICAP^®^ columns from Senova GmbH (Weimar, Germany) containing polyethylene filters with pore sizes of approximately 50 µm, a height of 5 mm, and a diameter of 5 mm were primed with ethanol in a desiccator to remove air bubbles from the filter. After washing the column twice with 500 µL of distilled water, 450 µL of nanomag^®^-D SPIO (Prod.#: 79-00-201; Micromod Partikeltechnologie GmbH, Rostock, Germany) was flushed through the column, followed by another washing step to ensure homogeneous MNP distribution and to remove unbound MNPs. The sample was dried at ambient conditions. Then the columns with the immobilized MNPs were measured at varying static field strength $H_{0}=\left(0,\ldots ,24\right)$ mT/*µ*_0_ in steps of 0.48 mT/*µ*_0_. Due to coil-current resistive heating, the temperature *T* in the measurement head was approximately 318 K. The first four nonlinear magnetic moment demodulation components from frequency mixing (both real and imaginary part) were stored: $f_{1}+n\cdot f_{2}$ with n=1,2,3,4. Background subtraction was performed using data from reference measurements without a sample, and phase correction for frequency-dependent phase shift inside the induction coil and amplification chain was performed.

### 2.2. Thermodynamic Langevin Model of a Magnetic Nanoparticle Ensemble

In the classical thermodynamic model description, the MNP sample can be described as an ensemble of noninteracting particles. (The validity of this assumption for our sample is assessed in Appendix A by an estimation of the dipole–dipole energy.) Neglecting surface effects, the saturation magnetic moment of a spherical particle with a core diameter $d_{c}$ is given by:

(2) $$\left|m_{p}\right|=m_{p}\left(d_{c}\right)=\frac{M_{s}\pi d_{c}^{3}}{6}$$

with *M_s_* denoting the saturation magnetization of the MNP. For simplicity, all particles are assumed to be spheres.

The total magnetic moment of the particle ensemble in thermodynamic equilibrium is calculated by averaging over a Boltzmann distribution of the orientations of the individual particle moments, yielding a dependence on the amplitude of the applied magnetic field $\left|H\right|=H$, which is governed by a Langevin function [16]:

(3) $$ℒ\;\left(\xi \right)=\coth\left(\xi \right)-\frac{1}{\xi }$$

with the dimensionless magnetic field variable:

(4) $$\xi =\frac{m_{p}\mu_{0}H}{k_{B}T}$$

and with temperature *T*, Boltzmann’s constant *k_B_* = 1.38 ×⋅10^–23^ J/K, and the permeability of vacuum *µ*_0_ = 4π ×⋅10^–7^ Vs/Am [17].

In the average over the particle ensemble, it has to be considered that the saturation magnetic moment *m_p_* of each particle depends on its diameter *d_c_*. Usually, particle ensembles exhibit a lognormal size distribution with a probability density function PDF(*d_c_*) given by [17]:

(5) $$\mathrm{PDF}\left(d_{c},d_{0},\sigma \right)=\frac{1}{\sqrt{2\pi }\cdot d_{c}\cdot \sigma }\cdot \exp\left(-\frac{\ln^{2}\left(d_{c}/d_{0}\right)}{2\sigma^{2}}\right)\;,$$

with the median diameter $d_{0}$ and the standard deviation σ of the diameters’ natural logarithm.

The total magnetic moment of the ensemble of *N_p_* particles is then calculated by integrating over the lognormal distribution:

(6) $$m_{tot}=N_{p}\int_{0}^{\infty }dd_{c}\cdot \mathrm{PDF}\left(d_{c}\right)\cdot m_{p}\left(d_{c}\right)\cdot ℒ\;\left(\frac{M_{s}\pi d_{c}^{3}}{6k_{B}T}\;\mu_{0}H\right)\;.$$

In our FMMD scheme [5], the particle ensemble is exposed to a two-frequency excitation with static offset magnetic field (see Equation (1)). Inserting Equations (1) and (2) into (6) yields:

(7) $$\begin{array}{l} m_{tot}=\frac{N_{p}M_{s}\pi }{6}\int_{0}^{\infty }dd_{c}\cdot \mathrm{PDF}\left(d_{c}\right)\cdot d_{c}^{3}\cdot \\ \cdot ℒ\;\left(\frac{M_{s}\pi d_{c}^{3}\mu_{0}}{6k_{B}T}\;\left[H_{0}+H_{1}\sin\left(2\pi f_{1}t\right)+H_{2}\sin\left(2\pi f_{2}t\right)\right]\right) \end{array}$$

As shown in [5], the nonlinearity of the magnetization curve gives rise to the emergence of intermodulation products *m*⋅*f*_1_ ± *n*⋅*f*_2_ of the total magnetic moment of the particle ensemble (with *m* and *n* denoting integers). In particular, the frequency mixing components *f*_1_ + *f*_2_, *f*_1_ + 2⋅*f*_2_, *f*_1_ + 3⋅*f*_2_ and *f*_1_ + 4⋅*f*_2_ appear. In the limit of small excitation amplitudes *H*_1_ and *H*_2_, these frequency mixing responses can be calculated with a Taylor expansion of Equation (3), yielding offset field dependencies of the mixing components proportional to the higher order derivatives of the Langevin function [5]. In the case of larger excitation amplitudes *H*_1_ and *H*_2_, the Taylor approximation is no longer valid and has to be replaced by the respective Fourier components of Equation (7). For instance, the average nonlinear moment response $m_{1}\left(d_{c}\right)$ at frequency *f*_1_ + *f*_2_ of one particle with diameter $d_{c}$ is given by:

(8) $$\begin{array}{l} m_{1}\left(d_{C}\right)=\frac{M_{s}\pi d_{c}^{3}}{6}\cdot \frac{2}{k}\sum_{i=0}^{k}\cos\left[2\pi \left(f_{1}+f_{2}\right)t_{i}\right]\cdot \;\\ \cdot ℒ\;\left(\frac{M_{s}\pi d_{c}^{3}\mu_{0}}{6k_{B}T}\;\left[H_{0}+H_{1}\sin\left(2\pi f_{1}t_{i}\right)+H_{2}\sin\left(2\pi f_{2}t_{i}\right)\right]\right) \end{array}$$

The factor 2/*k* normalizes the sum and accounts for the full-period average over sin^2^(..) which is ½. The sampling time steps *t_i_* should be chosen such that a sufficient number of samples is taken in one high frequency period. In our calculations, we took 10 steps in a period 1/*f*_1_, i.e., Δ*t* = *t_i_* – *t_i_*_–1_ = 0.1/*f*_1_, yielding sufficient numerical precision. Although in our experiments, the high frequency *f*_1_ was 485 times larger than the low frequency *f*_2_, it was sufficient to select *f*_1_ = 20*⋅f*_2_ for our numerical calculations. Thus, *k* = 200 was used.

In a similar fashion, the response component $m_{2}\left(d_{c}\right)$ at frequency *f*_1_ + 2⋅*f*_2_ is obtained from:

(9) $$\begin{array}{l} m_{2}\left(d_{c}\right)=\frac{M_{s}\pi d_{c}^{3}}{6}\cdot \frac{2}{k}\sum_{i=0}^{k}\sin\left[2\pi \left(f_{1}+2f_{2}\right)t_{i}\right]\cdot \;\\ \cdot ℒ\;\left(\frac{M_{s}\pi d_{c}^{3}\mu_{0}}{6k_{B}T}\;\left[H_{0}+H_{1}\sin\left(2\pi f_{1}t_{i}\right)+H_{2}\sin\left(2\pi \cdot 2f_{2}\cdot t_{i}\right)\right]\right)\;. \end{array}$$

Note the cos[..]/sin[..] alternation in the reference frequency term behind the sum symbol in Equations (8) and (9), which is due to the fact that with increasing order, the frequency mixing responses are alternately uneven (point-symmetric) and even (axisymmetric) functions. Components $m_{3}\left(d_{c}\right)$ and $m_{4}\left(d_{c}\right)$ are calculated similarly.

The total magnetic moment component $m_{n,tot}$ at the frequency mixing component *f*_1_ + *n*⋅*f*_2_ is then obtained by integration over the lognormally weighted particle ensemble:

(10) $$m_{n,tot}\left(d_{0},\sigma ,N_{p}\right)=N_{p}\int_{0}^{\infty }dd_{c}\cdot \mathrm{PDF}\left(d_{c},d_{0},\sigma \right)\cdot m_{n}\left(d_{c}\right).$$

Equation (10) constitutes our forward model for calculating the frequency mixing signals. The model contains just three fitting parameters, the lognormal distribution characteristics median diameter *d*_0_ and width *σ*, and the total number of particles $N_{p}$. The measured nonlinear magnetic moment components of nanoparticle samples at frequencies *f*_1_ + *n*⋅*f*_2_, *n* = 1, 2, 3, 4, were fitted with this model using the Levenberg–Marquardt least-squares algorithm.

### 2.3. Micromagnetic Monte Carlo (MC-)Simulation

The nonlinear particle relaxation dynamics in nonequilibrium conditions under the influence of an applied AMF, H**,** can be described by combined Néel–Brownian relaxation [13]. The Néel relaxation of the direction of the magnetic moment of a single MNP, $m_{p}$, is governed by the Landau–Lifshitz–Gilbert equation (LLG) [18]:

(11) $$\frac{dm_{p}}{dt}=\frac{\mu_{0}\gamma }{1+\alpha^{2}}\cdot \left(H_{\mathrm{eff}}\times m_{p}+\alpha m_{p}\times \left(H_{\mathrm{eff}}\times m_{p}\right)\right)$$

with the permeability of free space, $\mu_{0}$, the electron gyromagnetic ratio, γ, the damping parameter, α, and the effective field $H_{\mathrm{eff}}$**.** The Brownian rotation of the MNP easy axis, n, is described via the generalized torque, Θ, as follows [19]:

(12) $$\frac{dn}{dt}=\frac{\Theta }{6\eta V_{H}}\times n\;$$

with the carrier matrix viscosity, η, and the MNP hydrodynamic volume, $V_{h}=\pi /6\cdot d_{h}^{3}$, in which $d_{h}$ is the hydrodynamic particle diameter. Néel and Brownian relaxation are coupled in the internal particle energy:

(13) $$U=-\mu_{0}\cdot m_{p}\left(m_{p}\cdot H\right)-K\cdot V_{c}{\left(m_{p}\cdot n\right)}^{2}+\varepsilon_{IA}$$

where $m_{p}=\left|m_{p}\right|=V_{c}\cdot M_{S}$ (cf. Equation (2)) is the magnitude of the MNP magnetic moment, and $V_{c}=\pi /6\cdot d_{c}^{3}$ is the MNP core volume. The first term describes the Zeeman energy including the applied field H**.** The second term incorporates the anisotropy energy via the effective anisotropy constant, K, and under the assumption of uniaxial anisotropy and spherically shaped particles. The third term in Equation (13) includes magnetic dipole–dipole interaction. However, thermal energy, $\varepsilon_{\mathrm{therm}}$, dominates magnetic interaction energy by two orders of magnitude in our MNP samples, so that particle–particle interaction is negligible ($\varepsilon_{IA}\ll \varepsilon_{\mathrm{therm}}$). Please refer to Appendix A for a detailed estimation of the effect of magnetic dipole–dipole interaction energy that corroborates our assumption. Thermal fluctuations are included by adding the terms $H_{\mathrm{th}}$ and $\Theta_{\mathrm{th}}$, which are implemented as Gaussian-distributed white noise with zero mean values ($\langle H_{th}^{i}\left(t\right)\rangle =0$ and $\langle \Theta_{th}^{i}\left(t\right)\rangle =0$) and variances $\langle H_{\mathrm{th}}^{i}\left(t\right)H_{\mathrm{th}}^{j}\left(t’\right)\rangle =\left(2k_{B}\;T\cdot \left(1+\alpha^{2}\right)\right)/\left(\gamma m_{p}\alpha \right)\cdot \delta_{ij}\delta \left(t-t^{\prime }\right)$ and $\langle \Theta_{\mathrm{th}}^{i}\left(t\right)\Theta_{\mathrm{th}}^{j}\left(t’\right)\rangle =12k_{B}T\eta V_{H}\cdot \delta_{ij}\delta \left(t-t^{\prime }\right)$, respectively. Here T is the global temperature in the system. With this, the effective field and generalized torque read:

(14) $$H_{\mathrm{eff}}=-\frac{1}{m_{p}\cdot \mu_{0}}\cdot \frac{\partial U}{\partial m}+H_{th}=H+\frac{2K\cdot V_{c}}{m_{p}\cdot \mu_{0}}\cdot \left(m_{p}\cdot n\right)n+H_{th}$$

(15) $$\Theta =\frac{\partial U}{\partial n}\times n+\Theta_{th}=-2K\cdot V_{c}\left(m_{p}\cdot n\right)\left(m_{p}\times n\right)+\Theta_{th}$$

We apply the Stratonovic–Heun scheme to solve the system of coupled stochastic differential Equations (11) through (15) and implement a Monte Carlo method routine as described in our previous works [20,21,22]. The full source code is available as listed in the Data Availability Statement and its results are denoted as MC-simulations henceforth. Simulation parameters were chosen as listed in Table 1 with the MNP properties matching the experimentally determined values from Table 2. Furthermore, the damping parameter α was set to unity [23]. One thousand particles were simulated simultaneously and initialized with randomized directions of magnetization and easy axes for each MNP. The MNPs were then thermalized for one-fifth of the total number of time steps, N, before the AMF was applied. We used N=50,000 and averaged the magnetization over five independent simulation runs to achieve a good compromise between accuracy and acceptable computation time. The time step sizes were then 10 ns. The simulations were performed with the open-access Python code referenced in the Data Availability Statement. Calculations were carried out on a PC cluster consisting of 2 × CPU Intel Xeon E5-2687W, 3.1/3.8 GHz, with 8 clusters each and RAM 64 GB each. The typical calculation time for one offset field value was approx. 53 h.

To approximate experimental data for comparison, the excitation field parameters were chosen as $H_{1}=1$ mT/*µ*_0_, $f_{1}=40,000$ Hz, $H_{2}=16$ mT/*µ*_0_, $f_{2}=2000$ Hz, and the static field was varied from $H_{0}=\left(0,\ldots ,24\right)$ mT/*µ*_0_ with a step size of 1 mT/*µ*_0_.

## 3. Results

### 3.1. Experimental Results and Thermodynamic Langevin Model Fitting

**Figure 1:**
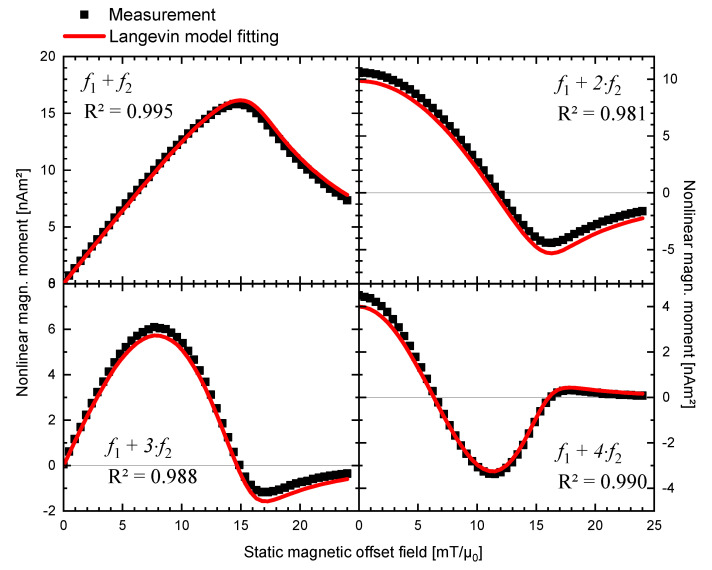
MNP nonlinear magnetic moment for dual frequency excitation at mixing frequencies $f_{LF}+n\cdot f_{HF}$ with n=1,2,3,4 from experimental measurement ($H_{1}=1.29$ mT/*µ*_0_, $f_{1}=30,534$ Hz, $H_{2}=16.4$ mT/*µ*_0_, $f_{2}=62.95$ Hz) and fitted with the Langevin model of Equation (10) with the same parameters.

The Langevin model (Equation (10), with inputs (8) and (9), see Section 2.2) was fitted to the measured real parts of the experimental data using the Levenberg–Marquardt least-square algorithm routine, whose results are plotted in Figure 1. Although the immobilized MNPs in the experimental setup are blocked in Brownian rotation, this step is justified because Langevin theory approximates the magnetization of the entire ensemble of MNP independently of the underlying mechanism of relaxation. The imaginary parts of the response signal were found to be two orders of magnitude weaker and were therefore disregarded. For all four demodulation components $f_{1}+n\cdot f_{2}$ with n=1,2,3,4, a very good agreement between experimental and simulated results was observed, confirmed by a coefficient of determination of $R^{2}>0.98$. Only for component *f*_1_ + *f*_2_ at high offset field did the simulation predict slightly higher values than measurement, whereas for *f*_1_ + 2⋅*f*_2_ and *f*_1_ + 3⋅*f*_2_, the simulation slightly underestimated measurements.

Fitting yielded the MNP material properties of median core diameter, $d_{c}$, and its log-normal distribution width, σ, which are listed in Table 2. The hydrodynamic diameter, $d_{h}$, and the concentration of the MNP were taken from the datasheet of the manufacturer.

### 3.2. Micromagnetic MC-Simulation Results

**Figure 2:**
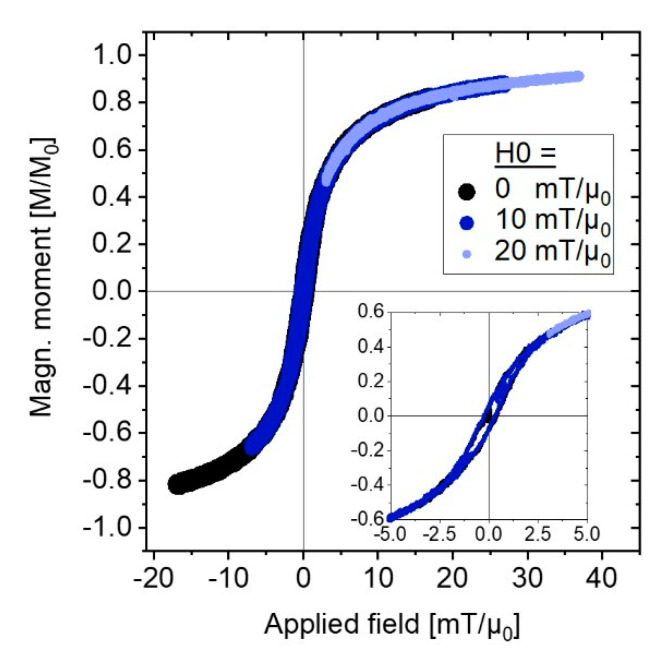
Exemplary magnetization curves (*M*(*H*)-loops) generated from micromagnetic MC-simulations for different static offset fields $H_{0}=\left(0,10,20\right)\;$mT/*µ*_0_. Inset shows magnification of small applied fields, revealing a slight opening of the loops.

We used the MNP material properties derived from fitting the Langevin model to the experimental data as described in the previous Section 3.1 (see Table 2) as input parameters for the micromagnetic MC-simulations. These simulations yield magnetization curves (*M*(*H*)-loops), which are shown for exemplary static offset fields in Figure 2.

The magnetization curves are (almost) identically overlapping, but shift towards the magnetization saturation of MNP as the static offset field, $H_{0}$, increases. The *M*(*H*)-loops are very slightly opened, revealing minor hysteresis area (see Figure 2, inset). For values of $H_{0}>\left(H_{1}+H_{2}\right)$, e.g., for $H_{0}=20$ mT/*µ*_0_ in Figure 2, the applied field is constantly keeping the MNPs in (almost) saturation and no hysteresis area is present.

**Figure 3:**
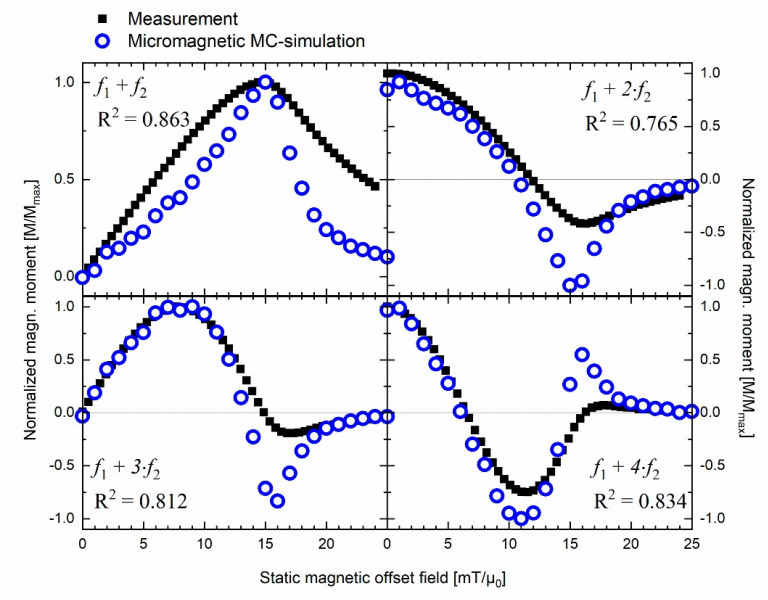
Normalized MNP nonlinear magnetic moment for dual frequency excitation at mixing frequencies $f_{1}+n\cdot f_{2}$ with n=1,2,3,4 comparing experimental results ($H_{1}=1.29$ mT/*µ*_0_, $f_{1}=30,534$ Hz, $H_{2}=16.4$ mT/*µ*_0_, $f_{2}=62.95$ Hz) and predictions from micromagnetic MC-simulations ($H_{1}=1\;\mathrm{mT}/µ0,\;f_{1}=40,000\;\mathrm{Hz},H_{2}=16$ mT/*µ*_0_, $f_{2}=2000$ Hz).

From the *M*(*H*)-loops, the first four demodulation components $f_{1}+n\cdot f_{2}$ were calculated following Equations (8) and (9). For comparison, the experimental measurement data and the MC-simulated data were normalized to their respective highest value, $M_{\max}$, and plotted alongside each other in Figure 3. The agreement between experimental and simulated data is acceptable, as confirmed by a coefficient of determination of $R^{2}>0.76$ for all four demodulation components. We observe for component *f*_1_ + *f*_2_ that MC-simulation consistently underestimates the measurement results. However, the peak at $H_{0}=15$ mT/*µ*_0_ coincides. For the mixing term *f*_1_ + 2⋅*f*_2_, MC-simulation underestimates the measurement results for $H_{0}<18$ mT/*µ*_0_ and $H_{0}>20$ mT/*µ*_0_ and shows a mismatch of over 50% for the peak value at $H_{0}=15$ mT/*µ*_0_. MC-simulation and measurement of component *f*_1_ + 3⋅*f*_2_ coincide for values up to $H_{0}=10$ mT/*µ*_0_ and again for $H_{0}\geq 20$ mT/*µ*_0_. However, around the peak at $H_{0}=16$ mT/*µ*_0_, MC-simulations overestimate experimental data by approx. 70%. For mixing term *f*_1_ + 2⋅*f*_2_, MC-simulations match experimental data, except around the peaks at $H_{0}=$11 mT/*µ*_0_ and $H_{0}=16$ mT/*µ*_0_.

### 3.3. Comparing Micromagnetic MC-Simulation Results and Thermodynamic Langevin Model Fitting

**Figure 4:**
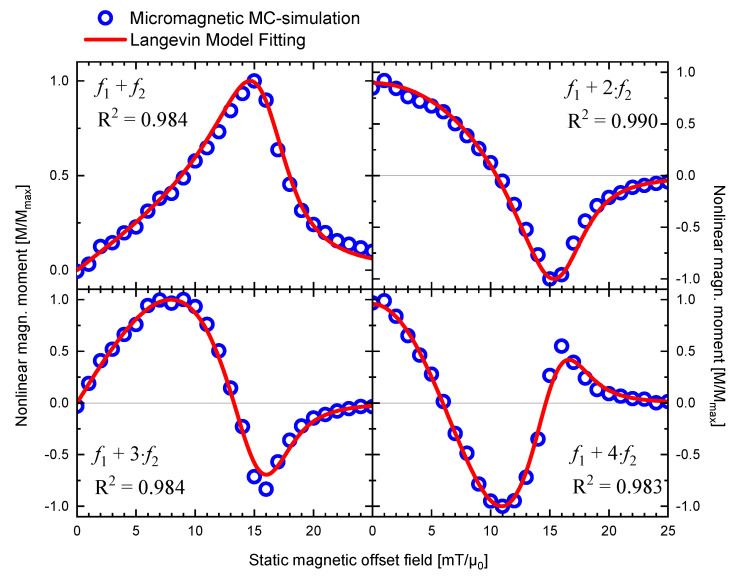
Normalized MNP nonlinear magnetic moment for dual frequency excitation at mixing frequencies $f_{1}+n\cdot f_{2}$ with n=1,2,3,4 from micromagnetic MC-simulations fitted with the thermodynamic Langevin model with fixed core diameter $d_{C}=7.813$ nm.

To test whether predictions from the Langevin model and MC-simulations show reproducible results, we fitted the Langevin model directly to the results from MC-simulation. We used the same input parameters for MC-simulations and Langevin model fitting of $H_{1}=1$ mT/*µ*_0_, $f_{1}=40,000$ Hz, $H_{2}=16$ mT/*µ*_0_, $f_{2}=2000$ Hz, and fixed the mean core diameter with $d_{c}=7.81$ nm and variable distribution parameter σ for the fitting. The results are plotted in Figure 4, confirming overall good agreement with a coefficient of determination of $R^{2}>0.989$ for all four demodulation components. From the qualitative comparison, one sees that Langevin model fitting and MC-simulations coincide, except for the secondary peaks for n=3 and n=4 (cf. Figure 4). The fitting yields a distribution width of σ=1.466. This is significantly different than the input parameters to MC-simulations (σ=0.346), questioning our hypothesis that assumes identical outputs from the Langevin model and MC-simulation. As we discuss in detail in the next section, we suspect the reasons for this lie with MNP properties that are not (yet) accurately represented in the modeling.

## 4. Discussion

**Figure 5:**
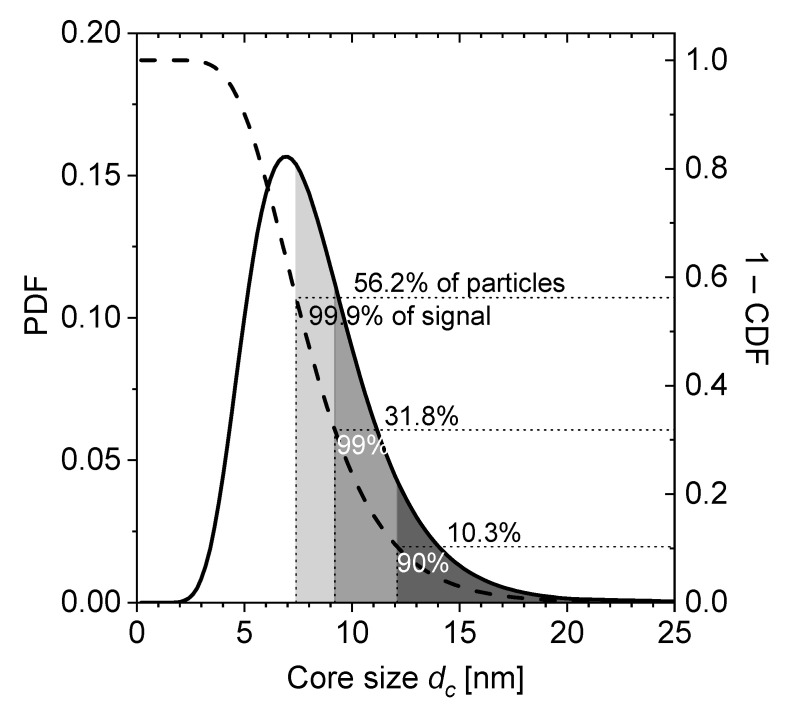
PDF of lognormal distribution (solid line) with *d*_0_ = 7.81 nm and *σ* = 0.346 and its reverse CDF, counted from large sizes (dashed line). The quantiles which yield 90% (99%; 99.9%) of the FMMD signal are shaded in dark grey (grey; light gray), they consist of 10.3% (31.8%; 56.2%) of particles on the large-sized tail of the distribution.

The Langevin model has been extensively applied to describe MNP magnetization for various applications such as diagnostic biosensing [25], Magnetic Particle Imaging (MPI) [26], and therapeutic Magnetic Fluid Hyperthermia (MFH) [27]. Its application to frequency mixing excitation seems therefore naturally reasonable. This assumption is fostered by our results reporting very good agreement between experimental data and Langevin model fitting ($R^{2}>0.98$; cf. Figure 1). The resulting fitting parameters ($d_{c}\approx 7.8$ nm and σ≈0.35; cf. Table 2) are furthermore in good agreement with literature values reporting a core diameter between $d_{c}\approx 7$ nm [28] and $d_{c}\approx 11$ nm [29] for nanomag^®^-D SPIO. The broad size distribution (with width σ>0.3) reflects a heterogeneous particle size with an effectively range of $d_{c}=\left(3-25\right)\;$nm, cf. Figure 5.

Nevertheless, the Langevin model is only valid for noninteracting, single particle ensembles with uniaxial anisotropy in thermodynamic equilibrium and (quasi)static magnetic fields [16]. Therein lies its major limitation, because the Langevin model cannot model an opening of *M*(*H*)-loops (hysteresis), which are, however, expected for AMF excitations at applied frequencies that approximate the inverse MNP relaxation time, $f\;\sim \;\tau^{-1}$, as the magnetic moment of each MNP begins to lag behind the driving AMF [2]. In contrast, micromagnetic MC-simulations provide new insight in the frequency mixing excitation of MNP by modeling the non-equilibrium dynamics of MNP by including thermal fluctuations: The MC-simulations reveal a minor hysteresis in the *M*(*H*)-loop of the MNP (cf. Figure 2), which is inaccessible via equilibrium Langevin theory. We attribute this minor hysteresis to the small portion of large particles, whose magnetic relaxation is (thermally) blocked by the volume (and therefore size cubed) dependent anisotropy barrier: $K\cdot V_{C}$.

This can be assessed by assuming an AC-field-dependent Brownian relaxation time of [30]:

(16) $$\tau_{B}\left(H_{\mathrm{AC}}\right)=\frac{\tau_{B}}{\sqrt{1+0.21\cdot \xi {\left(H_{AC}\right)}^{2}}}$$

and an AC-field dependent Néel relaxation time of [16]:

(17) $$\tau_{N}\left(H_{AC}\right)=\tau_{0}\cdot \exp\left(\frac{K\cdot V_{C}}{k_{B}\;T}\cdot {\left(1-\frac{H_{AC}}{H_{K}}\right)}^{2}\right)$$

where $H_{AC}$ denotes the AMF amplitude, $\tau_{B}=3\eta V_{H}/\left(k_{B}T\right)$ the field-independent Brownian relaxation time, $\xi \left(H_{AC}\right)=mH_{AC}/\left(k_{B}T\right)$ the reduced field parameter (cf. Equation (4)), $\tau_{0}=10^{-9}$ s the time constant, and $H_{K}=2K/{µ}_{0}M_{S}$ the (uniaxial) anisotropy field strength. From Equations (16) and (17), the effective relaxation time follows with:

(18) $$\tau =\frac{1}{\tau_{B}^{-1}+\tau_{N}^{-1}}.$$

Using the (mean) values from Table 1 and Table 2, and the experimental setup parameters with $H_{AC}=H_{1}+H_{2}\;\sim \;17\;$mT/*µ*_0_, one calculates $\tau \;\sim \;\tau_{0}\;\sim \;10^{-9}$ s so that for $f\;\sim \;\left(30-40\right)$ kHz the condition for the onset of hysteresis, $f\;\sim \;\tau^{-1}$, is not fulfilled. Because the measured imaginary parts of the mixing components are hundredfold weaker than the real parts, we can conclude that dissipation is indeed negligible. However, for the small portion of large particles with $d_{C}\;\geq \;20$ nm and naturally increasing the effective hydrodynamic diameter $d_{h}>20$ nm, and presumably decreasing effective anisotropy constant for these larger core particles, K ~ 5 kJ/m^3^ [31,32], the effective relaxation time increases by several orders of magnitude. The relaxation time for these large particles is dominated by the Brownian relaxation mechanism, fulfilling the condition $f\;\sim \;\tau^{-1}.\;$Consequently, the minor opening of the *M*(*H*)-loop observed in Figure 2 from MC-simulations is a direct contribution from these large size particles relaxing with Brownian rotation. This assumption could, however, not be verified experimentally in our current study, because the MNPs were immobilized due to sample preparation, blocking Brownian rotation. Nevertheless, Figure 2 and Figure 4 demonstrate the potential insight to be gained on the micromagnetic level in the underlying mechanisms in dual frequency excitation responses of MNP (see discussion regarding future experiments below).

Interestingly, these large size particles apparently also contribute most dominantly to the Langevin model fitting to MC-simulation data (cf. Figure 4): When we deliberately restrict the particle size range for Langevin model calculation to the largest portion of core sizes (i.e., limiting the minimum core size $d_{c}$), we find that 90% of the calculated signal is contributed from the particles with $d_{c}\;\geq \;12.1$ nm. Similarly, 99% of the signal stems from $d_{c}\;\geq \;9.2$ nm. With the reverse of the cumulative distribution function (CDF) of the lognormal distribution (see Equation (A3) in Appendix B), the corresponding size fractions (quantiles) of the distribution are calculated, beginning from the large-sized tail. This finding is visualized in Figure 5. The largest 10.3% of the particles contribute 90% of the FMMD signal. Furthermore, 99% of the signal is produced by the largest 31.8% of particles. In other words, this means that almost all of the FMMD signal originates from the large-sized tail of the size distribution.

This could also explain why the Langevin model can be fitted with very good agreement to MC-simulation data (cf. Figure 4; R>0.989), even though it is physically unable to model nonequilibrium dynamic relaxation processes: The mathematical fitting routine can force the fitting parameters ($d_{0},\;\sigma ,\;N_{p}$) to values outside the model’s range of validity (for further details see Appendix B). As the opening *M*(*H*)-loop and our relaxation time approximations (see above) suggest, thermodynamic equilibrium is not valid anymore for large particles $d_{C}\;\geq \;20\;$nm at frequencies $f\;\sim \;\left(30-40\right)$ kHz. Therefore, fitting the model to the experimental data could lead to unreliable distribution parameters.

For other frequency-dependent biomedical applications of iron-oxide MNPs, such as MPI [33,34] or MFH [32,35], an optimal core size of $d_{c}\;\sim \;25$ nm has been suggested from experiment and MC-simulation, stressing the importance of open hysteresis loops for signal generation. Our results from Figure 4 and Figure 5 could therefore indicate that an opening of the *M*(*H*)-loop is also favorable for generating strong signals in mixed frequency measurements. As mentioned above, our current sample preparation does not allow experimental verification of this assumption, because Brownian rotation is blocked for the immobilized MNP. Even more so, this assumption remains to be tested in future experiments using MNP with larger core sizes, ideally suspended and freely rotatable in solution. In future experiments, the question of whether the Brownian mechanism dominates the MNP relaxation mechanism could be further probed by suspending MNP in gelated matrices (e.g., agarose or poly-acrylamid gels), in which Brownian relaxation has been shown to be controllably blocked in MFH measurements with particles of the same size as used in the present study [36].

MC-simulations deviated quantitatively in intensity at the peaks by up to 70% from the experimental values (cf. Figure 3) when directly compared. The first reason for this could be that Brownian relaxation mechanism is blocked for immobilized MNPs in the experimental sample, while it adds to the signal in MC-simulations (see paragraph above). A second potential reason for deviations is that the parameters used for MC-simulation input slightly differ from the experimental parameters. Another reason could be that MNPs form clusters of a few particles in the experimental setting, which Dennis et al. consistently found for nanomag-D SPIO from small-angle neutron scattering (SANS) and transmission electron microscopy [37]. Clustering of MNPs has been suggested to lead to increased particle–particle interaction, changed effective anisotropy, and restriction of MNP rotatability [38,39,40]. Although the interplay of these clustering effects on MNP relaxation behavior is the subject of ongoing discussions [39,40,41,42], it is overall assumed to diminish the experimental signal intensity [43]. Both the rise of particle–particle interaction—although purposefully excluded from this study—and the influence of varying K could be investigated in future MC-simulations studies (e.g., by diffusion-limited colloidal aggregation [42]) to advance the understanding of its influence on mixed frequency excitation signal generation.

Finally, we are aware that our current study is limited by the extraction of input parameters for the MC-simulations from Langevin model fitting to experimental data (cf. Table 2). Future studies will complement these assumptions by experimental methods, e.g., measuring MNP core sizes via transmission electron microscopy (TEM) or the MNP magnetic core size distribution from magnetic measurement [44], in addition to determining the hydrodynamic diameters from dynamic light scattering (DLS) experiments [20], which will serve as input parameters to further validate our fitting and modelling.

## 5. Conclusions

With the current work, we present the first insights into dual-frequency magnetic excitation of MNP from interpreting experimental results in terms of both the thermodynamic Langevin model and nonequilibrium dynamic MC-simulations. In summary, we draw the following conclusions from our comparative study:

1. Both the Langevin model and MC-simulations reproduce the shape of the experimental signal satisfactorily (see Figure 1 and Figure 3). However, punctual deviations between experimental data and MC-simulations are observed (see Figure 3).
2. MC-simulations allow the investigation of the dynamic hysteresis (*M*(*H*))-loop during AMF excitation, revealing a minor opening (cf. Figure 2). This opening is attributed to the small portion of large, thermally blocked particles.
3. Langevin model fitting suggests that 90% of the experimentally detected FMMD signal intensity is generated by the largest 10% of the particles (cf. Figure 5).
4. For the large particles ($d_{c}>20$ nm) which dominate the FMMD signal, relaxation cannot be neglected. However, this effect is not included in our Langevin model. We suspect this is the reason for observed deviations between the Langevin model and MC-simulations.

We are aware that these findings raise more questions and could serve as the starting point for further investigations. Based on these conclusions we propose the following respective future research directions:

- Complementary experimental methods should be used to derive MNP properties for more accurate input in the MC-simulations (to address and remedy point 1, above). From this, we also plan to further increase our predictive accuracy in the future by coordinating simulation and experimental parameters to be identical (both materials properties and field parameters).
- Experimental sample preparation should be expanded to allow—ideally gradually controllable—Brownian rotation of MNPs (e.g., in poly-acrylamide gels) in order to precisely analyze the role of the Brownian relaxation mechanism for signal generation (see also next point below).
- Furthermore, MC-simulations could be expanded by including magnetic particle–particle interaction effects and the inclusion of clustering-effects of MNPs. This should be investigated with special regard to point 3 (above), because magnetic interaction scales with increasing particle core size. Additionally, the influence of effective anisotropy could be studied systematically with MC-simulations. Finally, the dominant relaxation mechanism in dual frequency excitation could be further investigated by weighting Néel and Brownian relaxation mechanisms systematically in MC-simulations, and comparing the results to experimental findings.
- Point 4 (above) suggests the necessity for multi-theory approaches to interpret dual-frequency MNP excitation responses. Therefore, systematic parameter studies, e.g., varying MNP properties such as effective anisotropy, median core sizes and shell sizes, and AMF parameters ($H_{1}$ $H_{2}$, $f_{1}$, $f_{2}$), should be performed to generate a multi-parameter repository from MC-simulations. This could serve as a basis for a unified model to explain FMMD signal generation in the future. However, such a study must be well designed to ensure acceptable computational effort.

## Figures and Tables

**Table 1:** Simulation parameters applied in the micromagnetic simulation model.

Effective Anisotropy Constant	Saturation Magnetization ^1^ *M_S_*	Mass Density of Magnetite	Viscosity of Surrounding (Water)	Temperature
11 kJ/m^3^	476 kA/m	5.2 g/cm^3^	$8.9\times 10^{-4}$Pa⋅s	300 K

^1^ The literature value for bulk magnetite from [24] was used.

**Table 2:** Material properties of MNPs from fitting the experimental data with the Langevin model.

Core Diameter $d_{c}$	Log-Normal Distribution Width σ	Polydispersity Index (PDI)	Hydrodynamic Diameter ^2^ ${d_{h}}^{\;}$	Concentration ^1^
7.81 nm	0.346	0.127	20 nm	2.4 mg(Fe)/mL

^1^ The concentration c is taken from the datasheet of the manufacturer. The concentration in the filter might be smaller due to unbound particles being washed out undetected. ^2^ The hydrodynamic diameter *d_h_* is taken from the datasheet of the manufacturer.

**Figure A1:**
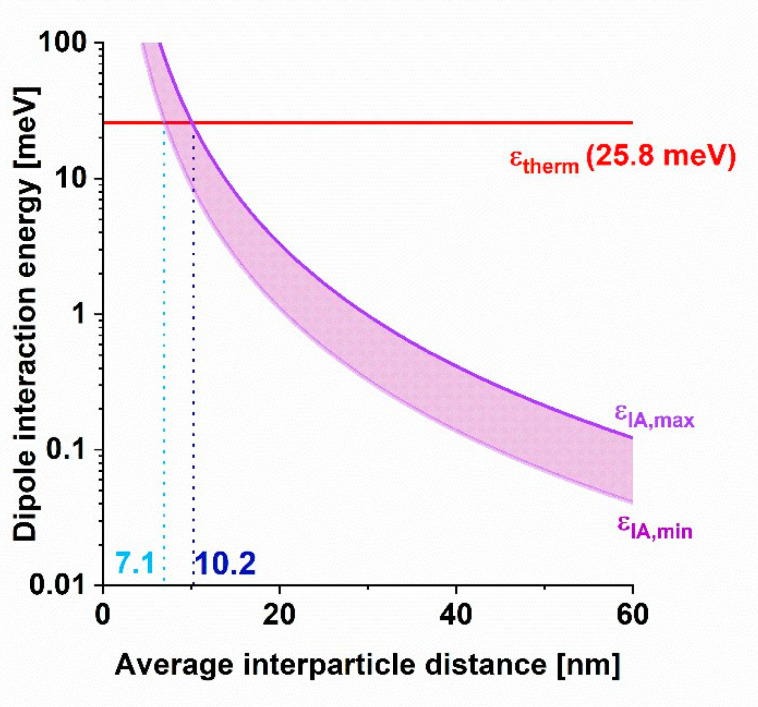
Estimated corridor for magnetic dipole–dipole interaction energy between neighboring MNPs (shaded) versus the average interparticle distance, estimated from Equation (A2). Thermal excitation energy is shown for reference.

**Figure A2:**
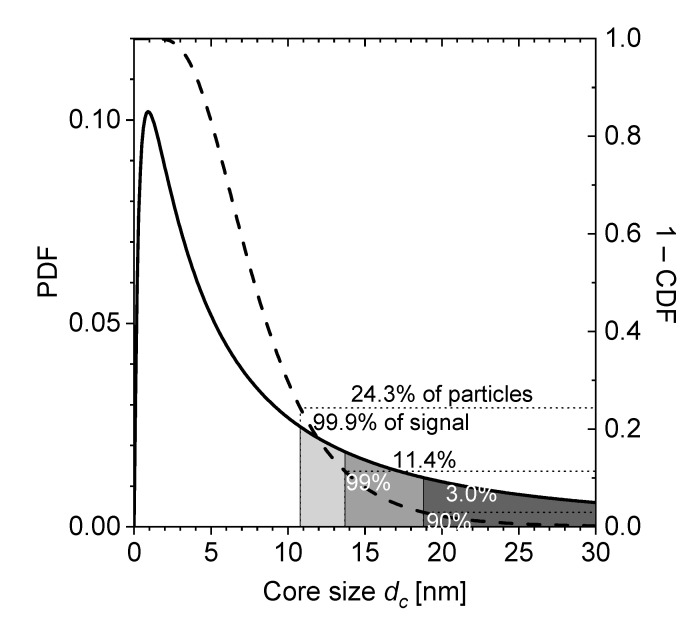
PDF of lognormal distribution (solid line) with *d*_0_ = 7.81 nm and *σ* = 1.466 and its reverse CDF, counted from large sizes (dashed line). The quantiles which yield 90% (99%; 99.9%) of the FMMD signal are shaded in dark grey (grey; light gray), they consist of 3.0% (11.4%; 24.3%) of particles on the large-sized tail of the distribution.

## Data Availability

Source code used for the nonlinear particle relaxation dynamics MC-simulations can be accessed at https://github.com/cshasha/nano-simulate.

## Appendix A. Estimating the Effect of Magnetic Dipole–Dipole Interaction Energy

The magnetic dipole–dipole interaction energy exerted on a single magnetic nanoparticle by *N* neighboring particles with interparticle distance vector $d_{i}$ can be described by [20]:

(A1) $$\varepsilon_{IA}=\sum_{\;i}\frac{\mu_{0}}{4\pi d_{i}^{3}}\cdot \left(\frac{3\cdot \left(m_{0}\cdot d_{i}\right)\cdot \left(m_{i}\cdot d_{i}\right)}{d_{i}^{2}}-m_{0}\cdot m_{i}\right)$$

For two neighboring particles with magnetic moments $\left|m_{0}\right|=\left|m_{i}\right|=m_{p}=V_{C}\cdot M_{S}$ (cf. Equation (2)), Equation (A1) can be simplified to the extremal energies:

(A2) $$\varepsilon_{IA,min}=\frac{\mu_{0}m_{p}^{2}}{4\pi d^{3}}\;\mathrm{and}\;\varepsilon_{IA,max}=3\frac{\mu_{0}m_{p}^{2}}{4\pi d^{3}}$$

Using Equation (A2) and the MNP properties from Table 2, we calculated a corridor for $\varepsilon_{IA}$ in dependence of the interparticle distance, shown in Figure A1. The thermal excitation energy of $\varepsilon_{therm}=k_{B}\cdot T=25.8\;$meV for T=300 K is also marked in Figure A1.

From the intersection of $\varepsilon_{\mathrm{therm}}$ and the corridor, the range of interparticle distances in which magnetic dipole–dipole interaction energy is of the same order of magnitude as the thermal excitation energy can be estimated with $d=\left(7.1-10.2\right)$ nm (see mark in Figure A1). In other words, for average interparticle distances d>10.2 nm, thermal excitation can be assumed to dominate magnetic interaction.

The average interparticle distance $d_{\mathrm{avg}}$ can be estimated from the number of particles in solution, z, by taking the inverse third root: $d_{\mathrm{avg}}=z^{-1/3}$. With $z=4.3\cdot 10^{15}\frac{1}{{\mathrm{cm}}^{3}}$ (taken from nanomag-D SPIO manufacturer’s datasheet) we estimate an average interparticle distance of $d_{\mathrm{avg}}\approx 61.5$ nm. From Figure A1 one can see that for such an average interparticle distance the thermal excitation energy is two orders of magnitude larger than magnetic dipole–dipole interaction energy, $\varepsilon_{therm}\gg \varepsilon_{IA}$, corroborating our assumption to neglect magnetic interaction between MNP for our calculations.

## Appendix B. Log-Normal Distribution Probability Density Function (PDF)

The log-normal distribution probability density function (PDF) for a general particle size d is defined as shown in Equation (5) with the median diameter $d_{0}$ and the standard deviation σ of the diameters’ natural logarithm. Integration over PDF from 0 to $d_{c}$ yields the cumulative distribution function (CDF) [17]:

(A3) $$\mathrm{CDF}\left(d_{c},d_{0},\sigma \right)=\int_{0}^{d_{c}}dd\cdot \mathrm{PDF}\left(d,d_{0},\sigma \right)=\frac{1}{2}+\left(1+\mathrm{erf}\left(\frac{\ln\left(d_{c}/d_{0}\right)}{\sqrt{2}\cdot \sigma }\right)\right)$$

Standard CDF cumulates particles starting from the small-sized tail of the distribution. For FMMD, large particles contribute most of the signal. To quantify their contribution, it is advantageous to consider the reverse cumulative distribution starting from the large size tail, i.e., 1—CDF. The exemplary log-normal distribution PDF(*d_c_*,*d*_0_,*σ*) and its reverse CDF using the parameters from Table 2 are plotted in Figure 5.

By fitting the Langevin model with fixed *d_c_* = 7.81 nm to the MC-simulation results, a significantly larger width parameter *σ* = 1.466 was obtained (see Section 3.3). The corresponding lognormal distribution and its reverse CDF are plotted in Figure A2. The FMMD signal calculated with this distribution is plotted in Figure 4. Analogously to the quantile analysis described in Section 4, the quantiles yielding 90%, 99%, and 99.9% of the FMMD signal were also determined for this wider distribution, as shown in Figure A2.

For this wider distribution, it was found that the largest 3.0% of the particles (with $d_{c}>18.8$ nm) contribute 90% of the FMMD signal. The largest 11.4% of particles (with $d_{c}>13.7$ nm) contribute 99% of signal. Almost all of the signal (99.9%) is generated by the largest 24.3% of the particles above 10.8 nm core size.
